# Evaluation of a rapid diagnostic test for measles IgM detection; accuracy and the reliability of visual reading using sera from the measles surveillance programme in Brazil, 2015

**DOI:** 10.1017/S0950268823000845

**Published:** 2023-08-04

**Authors:** Lenesha Warrener, Nick Andrews, Halima Koroma, Isabella Alessandrini, Mahmoud Haque, Cristiana C. Garcia, Aline R. Matos, Braulia Caetano, Xenia R. Lemos, Marilda M. Siqueira, Dhanraj Samuel, David W. Brown

**Affiliations:** 1 Public Health Microbiology Division, UK Health Security Agency (UKHSA), London, UK; 2 Immunisation and Vaccine Preventable Diseases Division, UKHSA, London, UK; 3 Laboratory of Respiratory Viruses, Exanthematics, Enteroviruses and Viral Emergencies (LVRE), Oswaldo Cruz Institute, FIOCRUZ, Rio de Janeiro, Brazil

**Keywords:** immunochromatographic test, ICT, immunoglobulin M, IgM, lateral flow, measles, rapid diagnostic test, RDT, rubeola, serology, surveillance, vaccine preventable disease

## Abstract

Laboratory-based case confirmation is an integral part of measles surveillance programmes; however, logistical constraints can delay response. Use of RDTs during initial patient contact could enhance surveillance by real-time case confirmation and accelerating public health response. Here, we evaluate performance of a novel measles IgM RDT and assess accuracy of visual interpretation using a representative collection of 125 sera from the Brazilian measles surveillance programme. RDT results were interpreted visually by a panel of six independent observers, the consensus of three observers and by relative reflectance measurements using an ESEQuant Reader. Compared to the Siemens anti-measles IgM EIA, sensitivity and specificity of the RDT were 94.9% (74/78, 87.4–98.6%) and 95.7% (45/47, 85.5-99.5%) for consensus visual results, and 93.6% (73/78, 85.7–97.9%) and 95.7% (45/47, 85.5-99.5%), for ESEQuant measurement, respectively. Observer agreement, determined by comparison between individuals and visual consensus results, and between individuals and ESEQuant measurements, achieved average kappa scores of 0.97 and 0.93 respectively. The RDT has the sensitivity and specificity required of a field-based test for measles diagnosis, and high kappa scores indicate this can be accomplished accurately by visual interpretation alone. Detailed studies are needed to establish its role within the global measles control programme.

## Introduction

Measles-containing vaccines have been in widespread use for more than 20 years as part of a global programme to control and eliminate measles. Between 2000 and 2016, annual mortality from measles decreased by 84% to an estimated 89780 deaths globally [1]. However, it continues to cause extensive morbidity and mortality in large areas of the world; being endemic in the Eastern Mediterranean, African and South-East Asia regions, where vaccine coverage is less than 80% in many member states. These regions account for approximately 98% of all global measles deaths [1]. Since 2017, the number of measles cases across the European Region has increased annually, with substantial outbreaks also occurring in other regions following measles virus importation into areas with under-vaccinated populations [1, 2]. This has been followed by the COVID-19 pandemic, which led to reduced reporting of measles cases. It has impacted severely on routine vaccination coverage leading to significant gaps in immunity and the number and scale of measles outbreaks has increased in 2022 [3]. In Brazil, successful vaccination programmes led to the elimination of measles in 2016, but this has not been maintained; with an epidemic that started in Amazonas in 2018, following an overspill of cases from Venezuela and has spread through the country since 2019 [4–6]. These epidemics emphasize the need for continued high-quality surveillance and rapid public health responses to limit transmission.

An essential component of surveillance is laboratory confirmation as diagnosis based on clinical signs alone is unreliable [7]. The basis of laboratory confirmation is the detection of measles-specific immunoglobulin M (IgM) antibodies in serum samples using commercially available enzyme immunoassays [7]. Although these tests are performed readily in laboratories with skilled staff, access to refrigeration and specialized equipment, maintaining the capacity to provide timely testing in resource-poor settings can prove difficult and kit supply may be intermittent in some regions. Shipment of samples to a centralized test facility may take several days, leading to a delay to obtain results [8].

The use of antibody detection, immunochromatographic, rapid diagnostic tests (RDTs) for the diagnosis of infectious diseases is becoming commonplace, particularly in low-resourced locations, as they are performed in a single incubation step at ambient temperature, without complex electrical equipment and their results can be interpreted visually, often within 15–20 minutes [9–13]. This allows rapid diagnosis to be made in the field or at facilities with minimal infrastructure, to increase diagnostic capacity, prompt implementation of public health responses and inform clinical management. We described recently a dipstick-formatted RDT for the detection of measles specific IgM antibodies in both serum and oral fluid (OF) specimens, which demonstrated good sensitivity and specificity [12, 13], but was not suitable for field use.

To improve useability and robustness of the test, the assay was redesigned as a housed RDT in which key measles-specific reagents and immunochromatographic strip were encased in a plastic cassette and the patient’s specimen is added via a sample port to initiate the test. We describe here the evaluation of the reformatted test device to investigate the accuracy of the measles IgM RDT for surveillance, using sera from a representative panel of suspected cases sent for measles investigation or confirmation to the WHO Measles Regional and National Reference Laboratory in Rio de Janeiro. Measles IgM RDT results were compared to those of the Siemens Enzygnost Anti-Measles Virus/IgM enzyme immunoassay (EIA), the standard reference assay. The accuracy of visual reading for future field use was also investigated.

## Materials and methods

### Serum samples

Ninety-seven sera collected from suspected measles cases, occurring between 2013 and 2015 in Brazil were investigated. Sera were received and tested by the State Public Health Laboratories, Ministry of Health, Brazil, as part of the routine national measles surveillance programme before referral to Instituto Oswaldo Cruz, FIOCRUZ (IOC) for measles confirmatory testing. Specimens were allocated a unique laboratory identifier on receipt by IOC, to decouple specimens from patient-specific information, prior to testing and storage at <−20°C in the routine laboratory archive.

Sera from measles cases were selected for inclusion by one of the study coordinators to ensure laboratory staff participating in testing were blind to previous results. Sera were chosen based on prior Siemens Enzygnost Anti-Measles Virus/IgM EIA corrected optical density (450 nm) (O.D.) measurements. Nineteen negative, <0.1 O.D., and 78 positive sera, with corrected optical density measurements distributed equivalently from the positive cut-off value of >0.2–2.409 O.D (450 nm), were included.

Sera from 28 cases of suspected dengue virus infection were included in the study to assess assay specificity. The sera were collected from Rio de Janeiro in 2015, at the time of a dengue virus outbreak and when the measles virus was not circulating in the city or state. All sera were stored at −20°C prior to this study.

### Enzyme immunoassays

All measles surveillance sera were tested using the Enzygnost Anti-Measles Virus/IgM and Enzygnost Anti-Measles Virus/IgG enzyme immunoassays in conjunction with the Supplementary Reagents for Enzygnost/TMB kit (Siemens Healthcare GmbH, Erlangen, Federal Republic of Germany, catalogue numbers: OWLI15, OWLN15, and OUVP17, respectively). Measles EIAs were performed and results were interpreted according to the manufacturer’s instructions, at the Laboratory of Respiratory Viruses, Exanthematics, Enteroviruses and Viral Emergencies, IOC, FIOCRUZ, Brazil.

The Enzygnost anti-measles virus/IgM EIA was configured in an indirect format, consisting of microtitre plate test wells coated with inactivated, cell culture-derived measles virus antigen and matched control wells coated with uninfected cultured cell antigen. Sera were diluted 1:20 in the provided diluent, mixed 1:1 with sheep anti-human IgG-Fc fragment absorbent solution and then incubated for 15 minutes before addition to the microtitre plate. One hundred and fifty microlitres of diluted sera, 1:41, and diluted anti-measles virus reference controls, 1:20, were added to paired measles antigen and control antigen wells and incubated at 37°C for 1 h. The plate was washed four times using an Asys Atlantis microplate washer (Biochrom Ltd., Cambridge, UK). Anti-human IgM-peroxidase conjugate, 100 μl per well, was added to the microplate and incubated for 1 h at 37°C. The plate was washed as described earlier and 100 μl tetramethylbenzidine dihydrochloride-hydrogen peroxide solution was added to all wells and incubated for 30 minutes, protected from light. Sulphuric acid (0.25 mol/L), 100 μl, was added per well. The absorbance of each well was measured at 450 nm using an ELX 808 spectrophotometric microplate reader (Biotek Instruments, Inc., Santa Clara, CA). The control well absorbance was subtracted from the test well absorbance for each kit control or specimen and multiplied by the manufacturer’s kit lot-specific correction factor. Sera with corrected absorbance values <0.100 were classified as negative, corrected absorbance values >0.200 were considered positive and corrected absorbance values ≤0.200 and ≥0.100 were interpreted as equivocal for the presence of measles-specific IgM.

The Siemens Enzygnost anti-measles virus/IgG EIA was presented in an indirect format, and was performed using identical procedural steps to the IgM assay described above, with the following exceptions; sera and anti-measles reference controls were diluted 1:230 and 200 μl added per well for testing, pretreatment of sera with sheep anti-human IgG-Fc fragment absorbent was not required and anti-human IgG-peroxidase conjugate provided in the IgG EIA kit by the manufacturer was used. Each result was expressed as a corrected optical density (450 nm) and interpreted using the same cut-off values as described for the IgM EIA. Quantitation of measles specific IgG in sera was also calculated, using kit lot-specific constants, *α* and *β*, provided by the manufacturer, in the following formula:

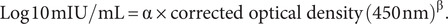



For the Dengue virus panel, the presence of dengue virus-specific IgM was detected using the Panbio Dengue IgM capture EIA (Alere S.A., Brazil, catalogue number: E-DEN01M), performed essentially as described by Berlioz-Arthaud and Gurusamy [14], at the Lacen Public Health Laboratory in Rio de Janeiro according to the manufacturer’s instructions. Sera from the Dengue virus panel were tested for the presence of measles-specific IgG and IgM, as described above, for inclusion in this evaluation.

### Training of laboratory staff

Six staff from the Laboratory of Respiratory Viruses, Exanthematics, Enteroviruses and Viral Emergencies, IOC, FIOCRUZ, Brazil, with limited or no experience of performing RDTs, were trained in the performance and result interpretation of the measles IgM RDT and operation of an ESEQuant LF Reader (Qiagen Lake Constance GmbH, Germany) prior to participation in the evaluation.

Training was delivered through a PowerPoint presentation of <1 h duration. Half-day laboratory training included demonstration of the RDT procedure, practice in visual result interpretation of RDTs by each staff member and demonstration in use of the ESEQuant LF Reader. A flow diagram of the main steps required to perform the RDT was provided as an aide memoire in the laboratory.

### Measles IgM RDT

The trial batch of measles IgM RDTs used in this study was prepared under subcontracted manufacture according to the specifications of the United Kingdom Health Security Agency (UKHSA).

**Figure 1.**
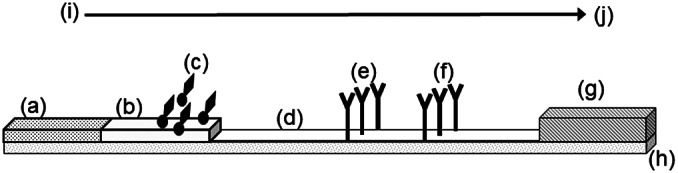
Schematic diagram of main components of the immunochromatographic test strip within the measles IgM RDT. Components of the measles IgM RDT are labelled as follows: (a) Sample pad and the location for specimen addition, (b) Conjugate release pad, (c) gold-conjugated measles antigen, (d) nitrocellulose membrane, (e) Test Line of immobilized anti-human IgM, (f) Control Line of immobilized monoclonal anti-measles antibody, (g) absorbent wicking pad, (h) adhesive, plastic backing card and directional arrow from (i) to (j), indicating the direction of reagent and specimen flow from sample addition pad (a), across the nitrocellulose membrane and terminating in the adsorbent pad (g)

The measles IgM RDT is an immunochromatographic assay encased in a plastic cassette. The components and principle of the assay are illustrated in Figure 1.

Affinity purified F(ab’)2 fragment goat anti-human IgM (109-006-129; Jackson ImmunoResearch Laboratories Inc., West Grove, PA) and mouse monoclonal anti-measles nucleoprotein antibody [13], each containing 0.08% w/v sodium azide (Sigma-Aldrich Co. Ltd., UK) were immobilized at the Test Line and Control Line positions of the nitrocellulose membrane and treated subsequently with a blocking solution. Colloidal gold nanoparticles (EM.GC40; BBI Solutions, Parkway, UK), conjugated to measles virus antigen [13], were dried into a conjugate release pad. The conjugate pad overlapped the analytical membrane on which the Test Line and Control Line capture reagents were located. The analytical membrane is visible in the viewing port of the device. A glass fibre pad was included at the proximal end to draw sample onto the test strip and an absorbent cellulose pad was placed at the distal end to absorb excess liquid. All solid phase components were attached to an adhesive plastic backing card prior to assembly into plastic cassettes. The RDTs were packaged individually in foil-mylar pouches, each containing a desiccant sachet and stored at <30 °C until testing was performed.

### Measles IgM RDT protocol

Evaluation of the measles IgM RDT was performed at ambient temperature within an air-conditioned laboratory; range: 19–25°C. A 100-fold dilution of each serum specimen was prepared by the addition of 5 μL of serum to 495 μL of oral fluid elution buffer containing 0.5%v/v Tween20, and mixed [7]. One hundred microlitres of diluted serum were added to the sample port of each RDT cassette and incubated at room temperature for 15 minutes. The RDTs were examined independently by three staff for the presence of Test and Control Lines, the perceived visual colour intensities of which were allocated a numerical score and recorded on a separate worksheet for each observer. The individual who performed the test set-up then recorded the relative reflectance measurements obtained for the Test and Control Lines using the ESEQuant LF Reader.

Five sera or less were tested in each test run and set-up rotated between five individuals to reduce operator bias. All six individuals performed visual Test and Control Line result interpretation independently.

### Visual scoring and interpretation of RDT results

The visual colour intensity of the Test and Control Lines were scored as follows:

Zero (0): no visible line observed = Negative, One (1): uncertain reaction/ incomplete line = Indeterminate, Two (2): complete line of pale pink colouration = weak positive, Three (3): moderate pink line = medium positive, Four (4): dark pink or red line = strong positive.

An RDT with visual scores of ≥2 at both the Test and Control Lines was interpreted as positive for the presence of measles-specific IgM in the specimen tested. A Test Line score of ‘1’ in the presence of a Control Line score of ≥2 was recorded as indeterminate and a Test Line score of ‘0’ with a Control Line score of ≥2 was scored as negative. A RDT was regarded as invalid if a consensus Control Line score of zero or one was assigned.

Worksheets with the visual Test and Control Line scores, for each serum sample tested in the RDT were collected from all observers and entered into an excel database for analysis.

### ESEQuant Lateral Flow Reader operation

Method parameters for the ESEQuant Lateral Flow (LF) Reader were defined using Lateral Flow Studio software (Qiagen Lake Constance GmbH, Germany). Expected positions of the Test Line and Control Line to enable gated measurement of each peak height relative to the background reflectance of the nitrocellulose membrane, expressed as millivolts (mV), and a baseline background reflectance of 30 mV were specified. This program was used throughout the evaluation.

The numerical relative reflectance measurements of the Test and Control Lines for each RDT were measured and recorded on a separate worksheet to visual interpretations. Numerical values were later entered into the excel database for analysis.

### Interpretation of ESEQuant LF Reader measurements

An RDT was defined as valid when the Control Line signal generated a relative reflectance measurement of ≥60 mV. An RDT was interpreted as being positive for measles-specific IgM when the Test Line measurement was ≥60 mV, and negative when the Test Line measurement was <60 mV, in the presence of a valid Control Line. The positive cut-off value (mV) for the automated RDT reader was established in the UK prior to this study, using a subset of measles and rubella sera tested previously [12].

### Statistical analysis

Consensus visual Test Line scores were determined for each RDT from the three independent observations. When two or more Test Line scores were the same, a consensus score equal to the majority value was assigned. When three different Test Line scores were recorded, the score closest to the average score was assigned. To calculate sensitivity and specificity of the RDT compared to the reference EIA, the consensus scores were categorized as positive, indeterminate and negative, as described above. The percentage agreement and kappa statistic, with 95% confidence interval (CI), were calculated to determine the interrater reliability, that is, the agreement between the visual Test Line score assigned by an individual observer and the consensus visual Test Line score, from zero to four [15]. Kappa analysis was repeated using three result interpretations: negative: Test Line = 0; indeterminate: Test Line = 1; and positive: Test Line ≥2.

To determine the agreement between the visual Test Line scoring by each observer and the numerical ESEQuant Test Line measurements, the ESEQuant Test Line measurements were assigned to five measurement ranges, as follows: Range Zero (0): <60 mV, Range One (1): 60–99 mV, Range Two (2): 100–299 mV, Range Three (3): 300–699 mV, and Range Four (4): ≥700 mV, for calculation of the kappa statistic. ESEQuant measurement ranges Two, Three, and Four were then combined to give a single range for positive measurements; ≥100 mV. The kappa statistic was recalculated to determine agreement between the visual score for each observer and the three ESEQuant measurement ranges; Negative Range: <60 mV, Weakly Reactive Range: 60–99 mV and Positive Range: ≥100 mV. Kappa scores were interpreted as moderate agreement 0.6–0.79, strong agreement 0.8–0.9, almost perfect 0.91–1.0 [15]. The agreement between the consensus visual Test Line score and the ESEQuant Test Line measurement ranges was also determined by recalculation of the kappa statistic.

## Results

### Characterization of serum specimens

Virus-specific IgM was detected in 93 of the 125 sera and was interpreted as confirmation of recent exposure to either measles, (*n* = 78), or dengue, (*n* = 15), viruses. One suspected dengue case, found to be IgM negative, gave an equivocal result in the measles IgM EIA. For the other 31 sera, measles IgG was detected in 18 of these; indicative of past measles infection or vaccination. The remaining 13 sera were negative (*n* = 11) or equivocal (*n* = 2) for measles IgG and the cause of rash fever illness was not determined.

## Measles IgM RDT results

### Consensus Visual Control Line interpretation

Visual Control Line scores from each of the three observers were compiled for all 125 RDTs. One hundred and twenty-four of 125 RDTs were interpreted as valid. Three observers each scored the Control Line of one RDT as ‘1’; indeterminate, leading to one invalid RDT out of 125 (0.8%). For two RDTs, one observer scored the Control Lines of two separate RDTs as ‘1’; indeterminate. However, on both occasions the other two observers scored the Control Lines as ‘2’; weak positive, resulting in consensus Control Line results of ‘2’, indicating both RDTS were valid for interpretation.

### ESEQuant LF Reader Control Line measurements

The range of Control Line measurements obtained using the ESEQuant LF Reader for the 125 RDTs was 68.18–1167.95 mV (mean: 860.54 mV), indicating all had passed the validity criteria of ≥60 mV established for the reader.

ESEQuant LF Reader measurements for the one RDT classified as invalid based on visual interpretation, and the two RDTs for which one of the observers scored the Control Line as indeterminate, were 68.18, 91.22, and 122.87 mV, respectively, which was consistent with a weak visual colour intensity of the Control Lines.

### Comparison of measles IgM RDT results with Anti-Measles Virus IgM EIA

Results obtained for the consensus visual score of measles IgM RDT on 125 sera compared to the reference EIA are summarized in Table 1. Visual scoring and interpretation of the consensus Test Line results of RDTs demonstrated a high level of sensitivity, 94.9% (74/78, 95% CI: 87.4–98.6%), specificity, 95.7% (45/47, 95% CI: 85.5-99.5%), and concordance, 95.2% (119/125) when compared with the Siemens Enzygnost Anti-Measles Virus/IgM EIA.

**Table 1.** Comparison of measles IgM RDT visual Test Line scoring with Siemens Enzygnost Anti-Measles Virus/IgM EIA results

	Measles IgM RDT results							
	RDT interpretation:	Positive			Indeterminate	Negative		
	RDT Test Line score:	4+	3+	2+	1+/−	0	Invalid	Total
Measles IgM EIA results	Positive	7	44	23	0	3	1	78
	Equivocal	0	0	1	0	0	0	1
	Negative	0	0	0	1	45	0	46
Total			75		1	48	1	125

A visual Test Line consensus score of ≥2 was interpreted as positive for measles in the presence of a valid Control Line. EIA, enzyme immunoassay; RDT, rapid diagnostic test.

Data comparing measles IgM RDT result interpretation using the ESEQuant LF Reader with the Siemens Enzygnost Anti-Measles Virus/IgM EIA are summarized in Table 2. Use of the single cut-off value of 60 mV to distinguish positive and negative RDT results demonstrated a high level of sensitivity, 93.6% (73/78, 95% CI: 85.7–97.9%), specificity, 95.7% (45/47, 95% CI: 85.5-99.5%), and concordance, 94.4% (118/125) when compared with the Siemens Enzygnost Anti-Measles Virus/IgM EIA.

**Table 2.** Comparison of measles IgM RDT result interpretations based on relative reflectance measured using the ESEQuant Lateral Flow Reader with the Siemens Enzygnost Anti-Measles Virus/IgM EIA

		Measles IgM RDT result interpretation based on ESEQuant LF Reader measurements		Total
		Positive (Test Line ≥ 60 mV)	Negative (Test Line < 60 mV)	
Measles IgM EIA results	Positive	73	5	78
	Equivocal	1	0	1
	Negative	1	45	46
	Total	75	50	125

A measles IgM RDT was interpreted as valid when the ESEQuant LF Reader reflectance peak height measurement at the Control Line was ≥60 mV. A relative reflectance peak height measurement of ≥60 mV at the Test Line was interpreted as positive for valid measles IgM RDTs. EIA, enzyme immunoassay; ESEQuant LF Reader, ESEQuant Lateral Flow Reader; mV, millivolts; RDT, rapid diagnostic test.

Concordant RDT and EIA results were obtained for 119 of 125 sera when RDTs were interpreted visually and for 118 sera when ESEQuant LF Reader interpretation was compared to EIA.

The one RDT interpreted as positive on visual inspection but negative using the ESEQuant LF Reader gave a Test Line measurement of 58.16 mV. This serum generated a weak positive result in the Siemens Enzygnost Anti-Measles Virus/IgM EIA, of 0.317 O.D (450 nm). During visual inspection, one of three observers had assigned a Test Line score of ‘1-indeterminate’, while the other two observers assigned a Test Line score of two, leading to an overall visual consensus of ‘2 – weak positive’. This sample was collected 4 days post onset of rash and had detectable anti-measles IgG, 4722 mIU/ml. This pattern of low or negative IgM and high titre of measles IgG soon after onset has been described previously for breakthrough infection in vaccinees [16, 17].

Three sera that were negative by RDT visual interpretation (Table 1), and positive in the EIA had corrected optical density (450 nm) measurements of 0.239, 0.322, and 0.354, just above the EIA positive cut-off value of 0.2 O.D (450 nm), suggesting measles specific IgM was present at a low level in the sera. The serum for which the RDT was interpreted as invalid based on visual result, was valid when measured using the ESEQuant LF Reader, as it gave a Control Line measurement of 68.18 mV. This serum also had low-level measles IgM EIA reactivity; 0.283 O.D (450 nm), an anti-measles IgG antibody titre of 5320 mIU/ml and was collected one-day post onset of symptoms. All four of these sera were interpreted as RDT negative based on ESEQuant LF Reader Test Line measurements of less than 60 mV; 0.00, 57.36, 33.05, and 0.78 mV, respectively.

The one measles IgM EIA negative serum interpreted as RDT indeterminate on visual examination gave a Test Line measurement of 63.1 mV using the ESEQuant LF Reader, indicating Test Line reactivity was weak. This specimen was collected on the day of rash onset and already had a detectable anti-measles IgG titre of 1575 mIU/ml.

The one measles IgM EIA equivocal serum, 0.168 O.D (450 nm), included in this study was positive in the measles IgM RDT on visual interpretation, with a Test Line score of ‘2’, and was also identified as positive by ESEQuant LF Reader, with a measurement of 211.1 mV. Although this serum was submitted as a suspected dengue infection, specific IgM was not detected by EIA. As measles IgG was detected and measles virus was not known to be circulating at this time, the cause of illness was unclear. Since it was equivocal in the reference measles IgM EIA we have treated this as a non-measles case for analysis. Hence, specificity of the RDT on suspected dengue cases was high; 96.4% (27/28). The RDT result may be due to non-specific reaction, as reported by other investigators [5], and was consistent with the specificity of Siemens Enzygnost Anti-Measles Virus/IgM EIA for this panel, also 96.4% (27/28).

### Agreement of individual observer visual results compared with visual consensus scores and with ESEQuant LF Reader measurements

The calculated agreement between visual interpretation of RDT results for each observer and the consensus visual score obtained from the three observers are shown in Table 3. Agreement of visual result interpretation for each individual observer, A–F, and the consensus interpretation was strong (average kappa = 0.82) when Test Line colour intensity was scored from zero; negative, to four; strong positive. The interrater reliability increased to near perfect (average kappa = 0.97) for visual interpretation when results were assessed using three categories only; negative, indeterminate and positive.

**Table 3.** Agreement of an individual observer’s visual Test Line scoring with consensus Test Line scoring from three observers

Observer identifier	No. RDTs read	Agreement between individual observers Test Line scoring and consensus Test Line result					
		Analysis using five visual Test Line scores; 0–4			Analysis using three visual result interpretations; negative, indeterminate, and positive		
		Percentage agreement	Kappa value	95% CI: kappa value	Percentage agreement	Kappa value	95% CI: kappa value
A	91	84.6	0.78	0.71–0.85	98.9	0.98	0.87–1.00
B	65	92.3	0.89	0.8–0.97	100.00	1.00	0.88–1.00
C	95	90.5	0.87	0.8–0.93	100.00	1.00	0.9–1.00
D	30	83.3	0.75	0.64–0.87	93.33	0.88	0.71–1.00
E	10	90.0	0.71	0.52–0.91	100.00	1.00	0.68–1.00
F	84	94.1	0.91	0.84–0.98	97.62	0.95	0.85–1.00
Observer average		89.1	0.82		98.3	0.97	

Consensus Test Line scores were determined from the combined visual interpretation from three independent observers. Each of the six staff members performing visual RDT interpretations was pseudonymized for publication and designated as A–F in the table. No., number; RDT, rapid diagnostic test; 95% CI, 95% confidence interval.

Agreement between each observer’s visual interpretation and the ESEQuant-measured result is shown in Table 4. Agreement was moderate (average kappa = 0.69) when ESEQuant Test Line reflectance measurements were divided into five numerical ranges, from zero; negative, to four; strong positive, but near perfect (average kappa = 0.93) when ESEQuant measurements were simplified to three result interpretations only. Agreement was also strong; kappa = 0.84, when the observer consensus was compared with ESEQuant measurements divided into five numerical ranges.

**Table 4.** Agreement of individual observer visual Test Line scoring and the visual Test Line consensus score with numerical ESEQuant LF Reader measurements

Observer identifier	No. RDTs read	Agreement between individual and consensus visual Test Line scoring and ESEQuant result ranges					
		Analysis using five ESEQuant result ranges; 0–4			Analysis using three ESEQuant result ranges; negative, weakly reactive, and positive		
		Percentage agreement	Kappa value	95% CI: kappa value	Percentage agreement	Kappa value	95% CI: kappa value
A	91	79.1	0.70	0.64–0.77	96.70	0.93	0.83–1.00
B	65	83.1	0.76	0.68–0.83	96.92	0.94	0.82–1.00
C	95	82.1	0.75	0.69–0.81	96.84	0.94	0.84–1.00
D	30	80.0	0.69	0.57–0.81	93.33	0.88	0.71–1.00
E	10	80.0	0.44	0.3–0.58	100.00	1.00	0.68–1.00
F	84	86.9	0.81	0.75–0.88	96.43	0.93	0.83–1.00
Test Line consensus	125	88.8	0.84	0.78–0.89	97.60	0.95	0.87–1.00
Observer average		81.9	0.69		96.7	0.93	

Each of the six staff members performing visual RDT interpretations was pseudonymized for publication and designated as A–F. No., number; RDT, rapid diagnostic test; 95% CI, 95% confidence interval.

**Figure 2.**
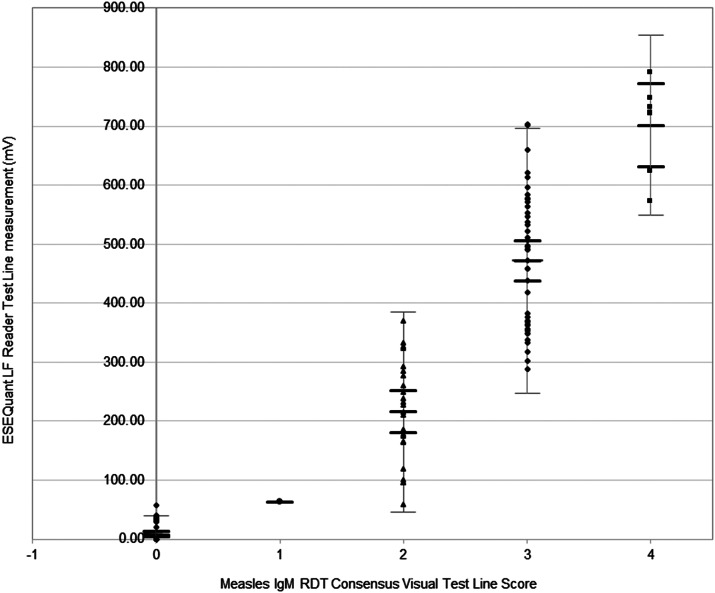
Distribution of ESEQuant LF Reader measurements compared to consensus visual scoring of test lines in the measles IgM RDT. Visual colour intensity of the Test Lines of valid RDTs was scored as follows: Zero (0): no visible line observed = Negative, One (1): uncertain reaction/ incomplete line = Indeterminate, Two (2): complete line of pale pink colouration = weak positive, Three (3): moderate pink line = medium positive, and Four (4): dark pink or red line = strong positive. The mean ESEQuant LF Reader Test Line measurement and 95% CI for each consensus visual Test Line scores are illustrated as horizontal black lines for the following visual scores: Score 0, 8.07 mV (95% CI, 3.56–12.59); Score 1, 63.1 mV; Score 2, 215.99 mV (95% CI, 180.16–251.81); Score 3, 471.98 mV (95% CI, 437.90–506.06), and Score 4, 701.26 mV (95% CI, 630.82–771.70). Upper and lower 95% sample population limits are illustrated as horizontal grey lines for each consensus visual Test Line score.

This indicated that some observers differed in perception of the colour intensity, leading to variation in scoring when multiple positive visual results, 2, 3, and 4, were compared to the ESEQuant Reader. However, the differences were far less frequent between positive and negative or when using a consensus of observers, as illustrated by the semi-quantitative correlation of visual Test Line consensus scoring with ESEQuant measurement in Figure 2.

The mean ESEQuant measurement obtained for each visual Test Line consensus score was: Score ‘0’, 8.07 mV (95% CI, 3.56–12.59); Score ‘1’, 63.1 mV; Score ‘2’, 215.99 mV (95% CI, 180.16–251.81); Score ‘3’, 471.98 mV (95% CI, 437.90–506.06), and Score ‘4’, 701.26 mV (95% CI, 630.82–771.70).

## Discussion

The aim of the study was to conduct a blinded evaluation of the performance of the cassette-housed measles IgM RDT compared to the current laboratory standard assay. The RDT gave accurate results, with high sensitivity and specificity, 94.9 and 95.7%, respectively. This level of performance is comparable to that of the Siemens EIA that has been used widely across the Global Measles and Rubella Laboratory Network (GMRLN) for the detection of measles IgM in serum samples [7]. The serum panel included samples from suspected cases of dengue virus infection to contribute to the investigation of specificity of the RDT, due to the similarity in clinical symptoms and potential for overlap in distribution in some regions. While the number of dengue virus-specific IgM-positive sera tested was limited, the initial indication was that specificity with this subset of sera was high. More extensive studies, with a range of infections causing rash fever, such as rubella and human parvovirus B19, are needed. The RDT device design is based on antibody capture, which was selected as the preferred format due to the demonstrated suitability for detection of the relatively low levels of IgM antibody found in oral fluid specimens [12, 13, 18, 19]. The use of oral fluids for antibody detection offers more flexibility in sampling and has been planned for future studies. Commercial availability of the assay would ensure both sustainable supply and enable cost-effective production. The study shows this RDT has a satisfactory level of performance and merits field and operational studies.

To exploit the full potential of the measles RDT for IgM detection in clinic and field settings in resource-poor locations, visual reading will be needed. We have investigated the accuracy of visual reading and demonstrated both a good correlation of visual reading between several individual observers and that individual visual readings also compared well with quantitative ESEQuant LF Reader measurements. This was demonstrated by the very high kappa scores achieved when classifying results as positive, negative and indeterminate, as would be used in a field setting. This indicated the test could be used reliably in the field, based on two independent visual readings, without the need or added cost of an automated reader. The laboratory staff trained to participate in the study had minimal or no prior experience of RDTs, but were able to use and read them reliably with very modest half-day of training.

Rapid Test Devices have been widely implemented in public health programmes for the diagnosis of infectious diseases such as HIV, malaria and dengue virus [9–11]. This has expanded the scale of testing and enabled the real-time confirmation of infection resulting in improved treatment and management pathways [11, 20]. Current measles surveillance programmes rely on the laboratory confirmation of cases by the detection of specific IgM in serum [7]. Despite the presence of the GMRLN, an accredited network of over 700 laboratories in 191 countries [21], operating to defined timelines for testing, in some resource-poor settings significant delays occur due to the need to transport samples to national laboratories and lack of kits [8]. With current EIAs, batch testing is the most cost-effective way to use kits and this too can lead to delay. The RDT described here, which was demonstrated to have a similar level of performance to the standard reference EIA, could improve the cost and flexibility of laboratory-based diagnosis, by enabling testing of only a few specimens at a time, and in response to demand. In its current format, utilizing the measles RDT to test serum samples has the potential to expand laboratory-based surveillance to a broader network. If adapted for use with capillary blood or oral fluid samples it could be used in field settings to facilitate a more timely diagnosis of measles resulting in the rapid implementation of control measures. Studies to evaluate the performance of an RDT designed for use with capillary blood and oral fluid are planned.

Many further questions remain regarding the operational use of the measles RDT to support its introduction into surveillance programmes. It is likely to be cost-effective, however, a similar rubella RDT will be needed to realize the potential to support integrated measles and rubella surveillance, as is conducted currently [7, 8]. The scale of testing needed is much less than HIV and malaria and the relative benefit of using measles RDTs in countries close to elimination versus in endemic settings and in a laboratory or field setting needs to be established. In countries with good measles control, real-time results have the potential to accelerate the measles public health response and reduce the burden of responding to suspected cases, which are later discarded. Utilization of the measles IgM RDT has the potential to transform measles and rubella surveillance.

## Data Availability

The data presented in this study are available on request from the corresponding author.
